# *In vivo* patch-clamp analysis of the antinociceptive actions of TRPA1 activation in the spinal dorsal horn

**DOI:** 10.1186/s12990-015-0021-6

**Published:** 2015-04-21

**Authors:** Manabu Yamanaka, Wataru Taniguchi, Naoko Nishio, Hiroshi Hashizume, Hiroshi Yamada, Munehito Yoshida, Terumasa Nakatsuka

**Affiliations:** Department of Orthopaedic Surgery, Wakayama Medical University, Wakayama, 641-8509 Japan; Pain Research Center, Kansai University of Health Sciences, Kumatori, Osaka 590-0482 Japan

**Keywords:** TRPA1, *In vivo* patch-clamp, Allyl isothiocyanate, Antinociceptive action

## Abstract

**Background:**

Transient receptor potential (TRP) channels are nonselective cation channels expressed in a variety of sensory structures, and are important molecular mediators of thermal, mechanical, cellular and chemical signals. We investigated the function of one key member of the TRP superfamily, TRPA1, in the spinal dorsal horn using *in vivo* patch-clamp recordings.

**Results:**

The application of allyl isothiocyanate (AITC), a TRPA1 agonist, significantly increased the frequency and amplitude of inhibitory postsynaptic currents (IPSCs; holding potential (V_H_) = 0 mV) as well as excitatory postsynaptic currents (EPSCs; V_H_ = −70 mV) in substantia gelatinosa (SG) neurons. The AITC-induced increases in EPSC frequency and amplitude were resistant to the Na^+^ channel blocker tetrodotoxin (TTX). In the presence of the glutamate receptor antagonists CNQX and AP5, AITC did not generate any synaptic activity. The AITC-induced increases in IPSC frequency and amplitude were abolished by TTX or glutamate receptor antagonists. Moreover, the duration of IPSCs enhanced by TRPA1 activation were significantly longer than those of EPSCs enhanced by activation of this channel in the spinal dorsal horn. AITC induced hyperpolarization of the membrane potential of SG neurons in the spinal cord but depolarized the membrane potential in the presence of TTX. Furthermore, we examined the effects of mechanical stimuli to the skin during TRPA1 activation in the spinal dorsal horn in normal rats in both voltage-clamp and current-clamp modes. In the peripheral tissue stimuli test, AITC significantly suppressed EPSCs evoked by pinch or air puff stimulation of the skin. In current-clamp mode, AITC significantly suppressed excitatory postsynaptic potentials (EPSPs) evoked by pinch stimuli.

**Conclusions:**

TRPA1 appears to be localized not only at presynaptic terminals on SG neurons, enhancing glutamate release, but also in the terminals of primary afferents innervating spinal inhibitory interneurons, which have synaptic interactions with SG neurons. This study offers further insight into the mechanisms underlying the possible antinociceptive actions of TRPA1 activation in the spinal dorsal horn. Our findings suggest that pharmacological activation of spinal TRPA1 channels may have therapeutic potential for the treatment of pain.

## Background

Transient receptor potential (TRP) channels are tetrameric, nonselective cation channels expressed in a variety of sensory structures. The TRP superfamily can be subdivided into seven families: TRPC, TRPV, TRPM, TRPP, TRPML, TRPA and TRPN [1-3]. Recently, it was shown that TRP channels are expressed at the peripheral terminals of primary afferent fibers. TRPA1, TRPM8 and TRPV1 are well-known molecular transducers of pungent agents, temperature, pain, lipids, acids, shear stress and inflammatory nociceptive signals [4-7]. TRPA1 is activated by noxious cold temperature, reactive oxygen species (ROS) and pungent natural compounds in mustard oil, cinnamon oil, ginger and garlic [8-13]. TRPA1 is found in a subset of primary sensory neurons where it is coexpressed with noxious heat-sensing TRPV1, but not non-noxious cool-sensing TRPM8 [13,14]. It has been found that TRP channels are also localized to the central terminals of primary afferent fibers in the spinal cord; and it is thought that TRP channels in the spinal cord are activated by various endogenous factors. However, the physiological role of spinal TRP channels remains unknown.

Our research group previously examined the function of TRP channels in the dorsal horn of rat spinal cord slices using whole-cell patch-clamp recordings. We found that ROS enhance excitatory synaptic transmission in dorsal horn neurons by activating TRPA1 and TRPV1 channels [12]. We have also reported that the activation of TRPA1 channels facilitates excitatory synaptic transmission in substantia gelatinosa (SG) neurons in the adult rat spinal cord and enhances glutamate release by direct Ca^2+^ entry through TRPA1 channels in nerve terminals [14]. These findings suggest that TRP channels in the dorsal horn of the spinal cord enhance nociceptive transmission. To date, it has been reported that four TRP channels, TRPA1, TRPM8, TRPV1 and TRPV4 are involved in neuropathic pain [15-18]. However, it was recently reported that intrathecal injections of N-acetyl-*P*-benzoquinoneimine and *P*-benzoquinone, which are metabolites of acetaminophen, activate the TRPA1 receptor and have antinociceptive effects [19]. Therefore, TRPA1 may be a promising pharmacological target for the development of new analgesics. In this study, we focus on the TRPA1 channel and investigate its role in pain transmission in the spinal dorsal horn using *in vivo* patch-clamp recordings.

## Results

Rats used in this study remained in a stable condition for over 10 h, comparable to previous experiments using an artificial ventilator. Whole-cell patch-clamp recordings were made from 182 SG neurons. All neurons studied had membrane potentials more negative than −50 mV. All SG neurons tested exhibited excitatory postsynaptic currents (EPSCs) at a V_H_ of −70 mV, and no inhibitory postsynaptic currents (IPSCs) were observed because the reversal potential for IPSCs was near −70 mV [20,21]. Furthermore, SG neurons exhibited IPSCs at a V_H_ of 0 mV, and no EPSCs were observed because the reversal potential for EPSCs was near 0 mV [21].

### The effect of TRPA1 activation on excitatory synaptic transmission

**Figure 1:**
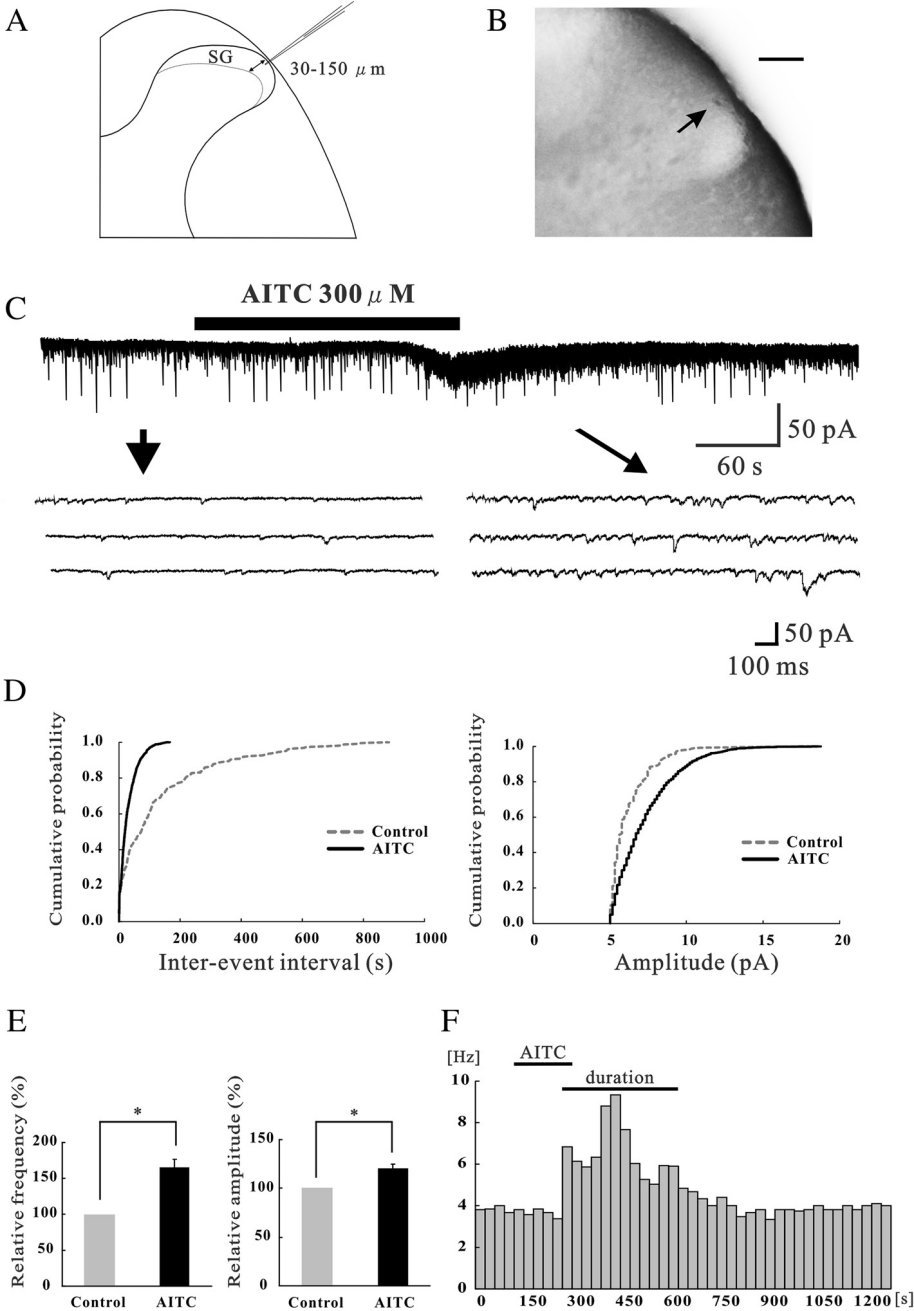
Identification of SG neurons and the action of AITC on excitatory synaptic transmission in SG neurons. **(A)** Schematic drawing of the transverse spinal cord slice and of a recording electrode. Recordings were made from cells at a depth of 30 – 150 μm (shown by arrows) from the surface of the spinal cord. **(B)** Transverse slices of the spinal cord at the level of L5. A recorded cell was identified with an intracellular injection with neurobiotin (shown by an arrow). After recording synaptic responses, the spinal cord was fixed and then cut into 500 μm thick slices. Scale bar is 100 μm. **(C)** Continuous chart recording of glutamatergic EPSCs before and during the action of AITC (300 μM; top). Three consecutive traces of EPSCs are shown in an expanded scale in time, before (bottom left) and under the action of AITC (bottom right). Note, a slow inward current that is accompanied by increases in EPSC frequency and amplitude (top). **(D)** Cumulative distribution of the inter-event interval (left) and amplitude (right) of EPSCs in control (dotted line) and during (continuous line) the action of AITC. AITC shifted the inter-event interval and amplitude to a shorter and a larger one (same neuron as in Figure 1C). **(E)** Summary of EPSC frequency (left) and amplitude (right) under the action of AITC (n = 68) relative to control. In this and subsequent figures, vertical or horizontal lines accompanied by bars indicate S.E.M. Statistical significance between data is indicated by an asterisk; *P < 0.05. **(F)** The frequency of EPSCs following the application of AITC plotted against time. Each bar indicates data calculated from the EPSCs measured for 30 s (same neuron as in Figure 1C). “Duration” represents the period when the frequency increases more than 20% of the control.

Neurons were recorded at a regular depth of 30–150 μm measured from the dorsal surface of the spinal cord to the point of contact with the cell. This distance was identified to be within the SG using transverse slices obtained from the spinal cords of 5-week-old rats at the same lumbar level. *In vivo* patch clamp recording was performed on a cell at a regular depth of 30–150 μm measured from the point of contact with the cell to the dorsal surface of the spinal cord (Figure 1A) [22,23]. We confirmed that neurons were located within the SG (Figure 1B) using neurobiotin staining.

**Figure 2:**
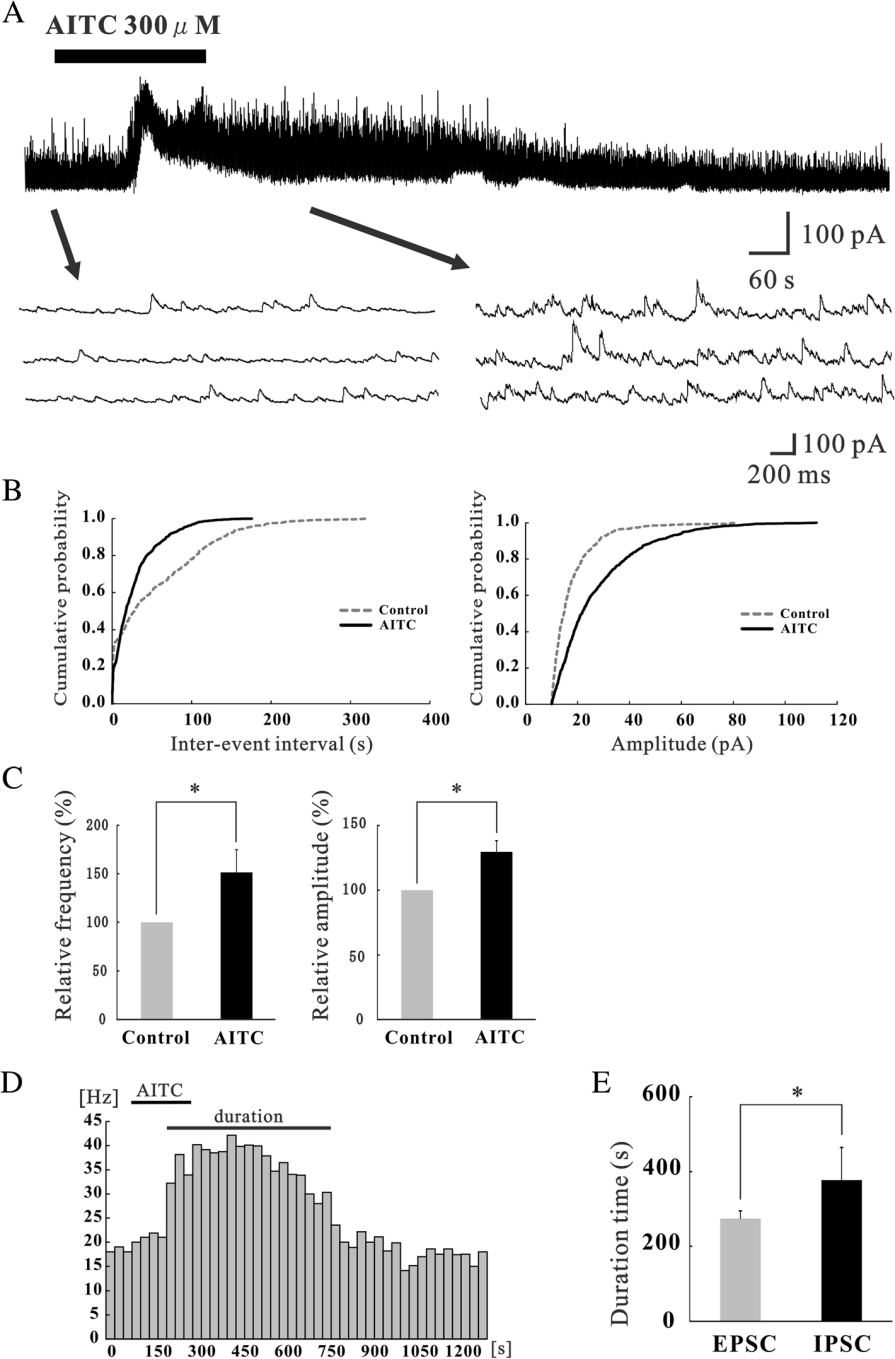
Actions of AITC on inhibitory synaptic transmission in SG neurons. **(A)** Continuous chart recording of IPSCs before and during the action of AITC (300 μM; top). Three consecutive traces of IPSCs are shown in an expanded scale of time, before (bottom left) and under the action of AITC (bottom right). The holding potential (V_H_) used was −0 mV. **(B)** Cumulative distribution of the inter-event interval (left) and amplitude (right) of IPSCs in the control (dotted line) and during (continuous line) the action of AITC. AITC shifted the inter-event interval and amplitude to a shorter and a larger one (same neuron as in Figure 2A). **(C)** Summary of IPSC frequency (left) and amplitude (right) under the action of AITC (n = 9) relative to control. **(D)** The frequency of IPSCs following application of AITC plotted against time. Each bar indicates data calculated from IPSCs measured for 30 s (same neuron as in Figure 2A). “Duration” represents the period when frequency increases more than 20% of the control. **(E)** Summary of average duration of IPSCs and EPSCs evoked by AITC (n = 5). *P < 0.05.

To assess the impact of TRPA1 channel activation on excitatory synaptic transmission, we used allyl isothiocyanate (AITC), the principal pungent compound in mustard oil and wasabi, as a selective TRPA1 agonist. Superfusing AITC (300 μM) for 3 min resulted in a significant increase in the frequency and amplitude of EPSCs in 36 of 68 neurons recorded (52.9%); this effect was accompanied by a slow inward current (>5 pA) in 32 of the 36 neurons (88.9%), as shown in Figure 1C. When measured for 30 s in the presence of AITC, the average increase in EPSC frequency and amplitude were 164.3 ± 17.6% and 120.5 ± 4.3% (n = 68), respectively (Figure 1E). Here, we show the mean peak frequency and amplitude of enhanced EPSCs by AITC in recorded neurons, including both responsive and non-responsive neurons. Figure 1D demonstrates the effects of AITC (300 μM) on the cumulative distribution of the inter-event interval and amplitude of EPSCs. AITC increased the proportion of EPSCs having a shorter inter-event interval (*p* < 0.05) and a larger amplitude (*p* < 0.05) when compared with the control (same neuron as in Figure 1C). Figure 1F demonstrates the frequency of EPSCs following application of AITC plotted against time. Each bar indicates data calculated from the EPSCs measured for 30 s (same neuron as in Figure 1C). We defined the “duration” as the period when the frequency increased by 20% compared with the averaged control (Figures 1F and 2E). The average duration of EPSCs was 274.2 ± 20.8 s in the presence of AITC.

### The effect of TRPA1 activation on inhibitory synaptic transmission

AITC (300 μM) robustly increased IPSC frequency and amplitude in 7 of the 9 neurons recorded (77.7%) (Figure 2A). When measured for 30 s in the presence of AITC, the average increase in IPSC frequency and amplitude, including both responsive and non-responsive neurons, were 151.5 ± 23.3% and 129.6 ± 8.6% (n = 9), respectively (Figure 2C). Figure 2B demonstrates the effects of AITC (300 μM) on the cumulative distribution of the interval and amplitude of IPSCs. AITC increased the proportion of IPSCs having a shorter inter-event interval (*p* < 0.05) and a larger amplitude (*p* < 0.05) when compared with control (same neuron as in Figure 2A). Figure 2D demonstrates the frequency of IPSCs following application of AITC plotted against time (same neuron as in Figure 2A). Interestingly, IPSCs induced by AITC had a significantly longer duration (average; 377.1 ± 87.8 s) compared with EPSCs (average; 274.2 ± 20.8 s) induced by AITC (Figure 2E).

### Characterization of EPSCs and IPSCs induced by AITC

**Figure 3:**
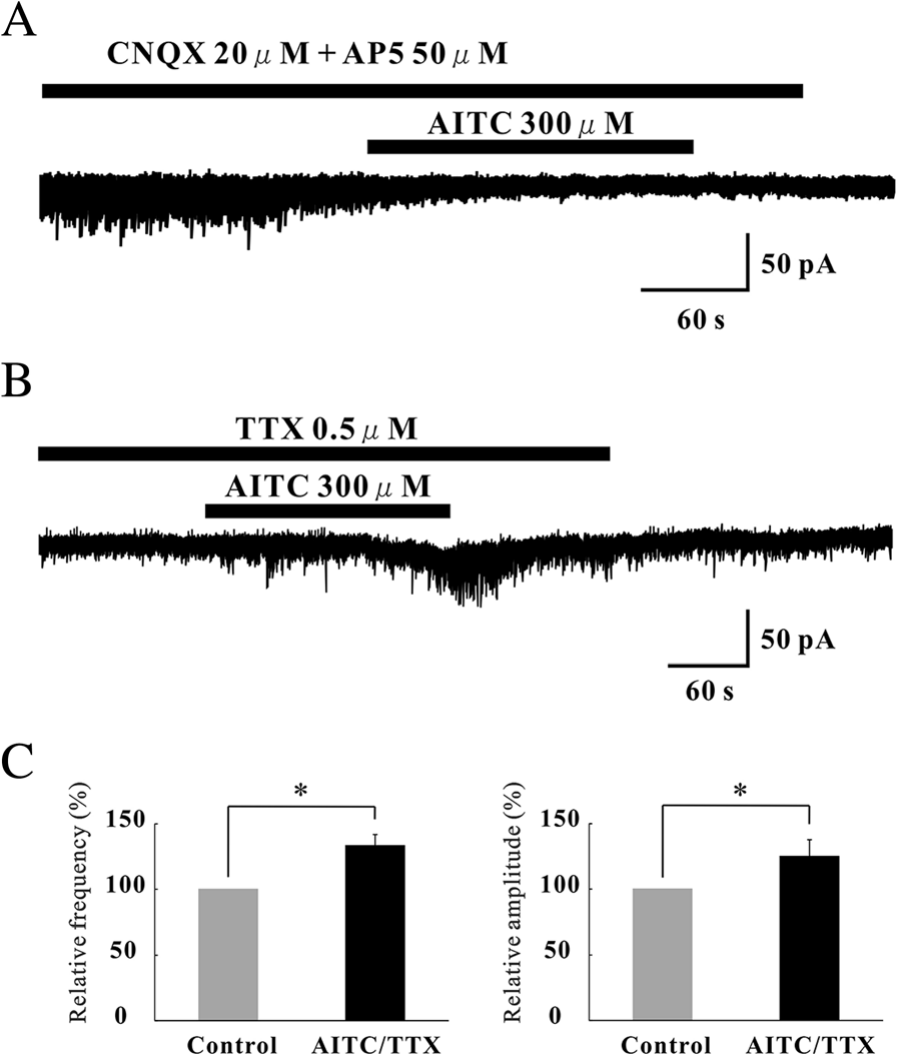
Characterization of EPSCs induced by AITC. **(A)** Action of AITC (300 μM) on EPSCs in the presence of CNQX (40 μM). CNQX blocked EPSCs not only in the absence of AITC but also under its action. Traces of EPSCs are shown in an expanded scale in time, control (bottom left) and under the action of CNQX (bottom right). **(B)** A continuous chart recording of glutamatergic miniature EPSCs in the presence of TTX (0.5 μM) before and under the action of AITC (300 μM). **(C)** Summary of EPSC frequency (left) and amplitude (right) under the action of AITC (n = 5) relative to control.

**Figure 4:**
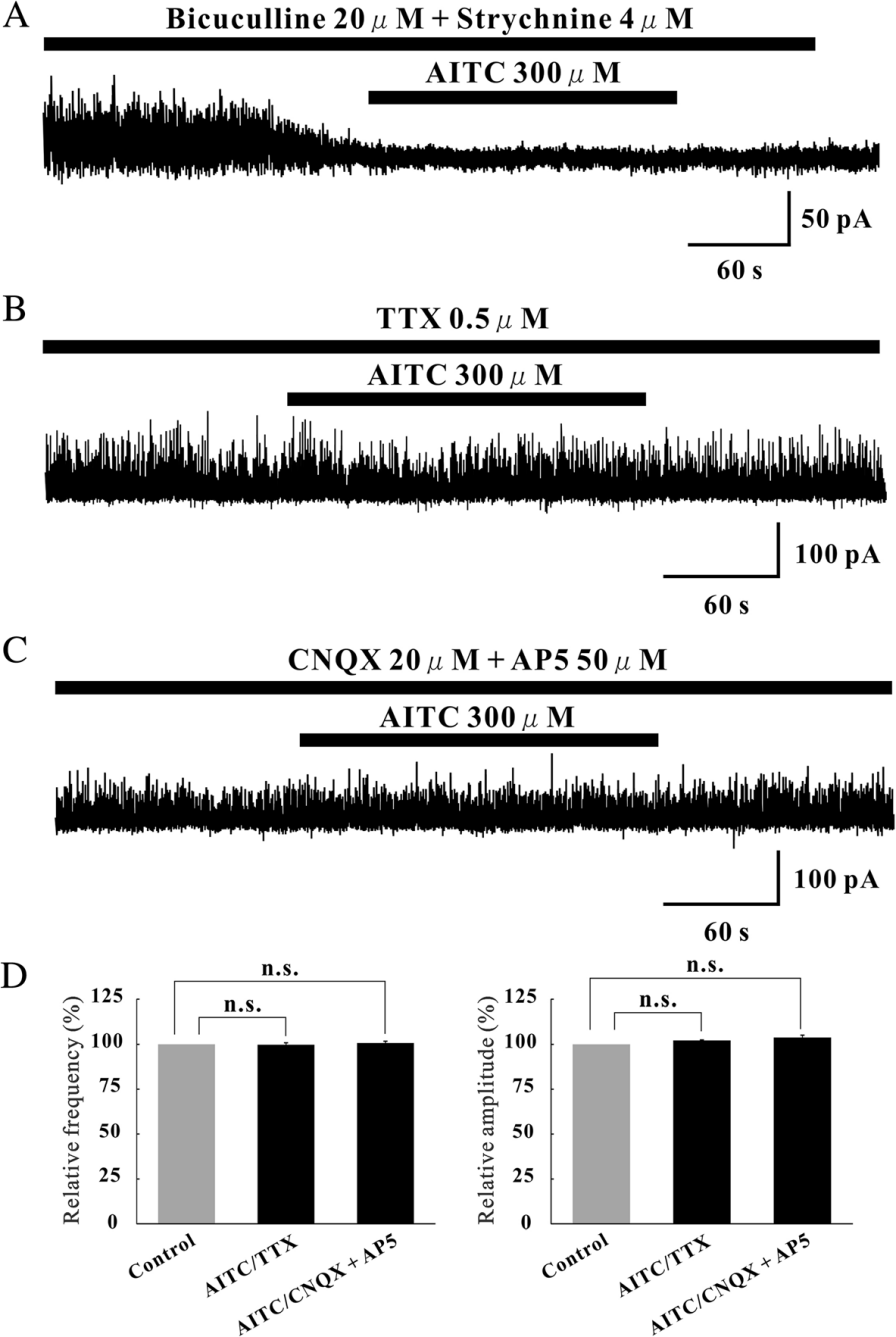
Characterization of IPSCs induced by AITC. **(A)** Action of AITC (300 μM) on IPSCs in the presence of both bicuculine (20 μM) and strychinine (4 μM). Both bicuculine and strychinine completely blocked EPSCs not only in the absence of AITC but also under its action. Traces of IPSCs are shown in an expanded scale in time, control (bottom left) and under the action of both bicuculine and strychinine (bottom right). **(B)** A continuous chart recording of IPSCs before and during the action of AITC (300 μM) in the presence of TTX (0.5 μM). TTX abolished the AITC-induced increases in IPSC frequency and amplitude. **(C)** A continuous chart recording of IPSCs before and during the action of AITC in a mixture of CNQX (20 μM) and AP5 (50 μM). The AITC-induced increase in IPSC frequency and amplitude was attenuated in the presence of the drugs. **(D)** Summary of EPSC frequency (left) and amplitude (right) under the action of AITC in the presence of TTX (n = 5) and AITC in the presence of CNQX + AP5 (n = 5), relative to those in the control. Vertical lines accompanied by bars show SEM. Statistical significance between data shown by bars is indicated by an asterisk; *p = 0.05. n.s., Not significant.

The AMPA receptor antagonist 6-cyano-7-nitroquinoxaline-2,3-dione (CNQX) and the NMDA receptor antagonist D-(−)-2-amino-5-phosphonopentanoic acid (AP5) were tested in neurons in which AITC increased EPSC frequency and amplitude. CNQX (20 μM) and AP5 (50 μM) suppressed EPSCs not only in the control, but also under the action of AITC (Figure 3A), indicating that AITC caused a robust glutamate release onto SG neurons. We further investigated whether AITC-sensitive terminals were monosynaptically connected with SG neurons. This was done by determining the effects of AITC (300 μM) on EPSCs in the presence of TTX (0.5 μM). Figure 3B demonstrates that AITC markedly increased both EPSC frequency and amplitude in the presence of TTX (138.9 ± 7.8% and 125 ± 12.8%, n = 5) (Figure 3C). However, AITC-induced IPSCs were blocked by strychnine (a glycine-receptor antagonist) or bicuculline (a GABA_A_-receptor antagonist) in all 5 neurons examined (Figure 4A). When AITC (300 μM) was perfused in the presence of TTX (0.5 μM), IPSC frequency and amplitude were unaltered (102.2 ± 4.8% and 99.8 ± 0.33% of control respectively, n = 5) (Figure 4B). Moreover, AITC (300 μM) did not affect IPSC frequency and amplitude in a mixture of CNQX (20 μM) and AP5 (50 μM) (103.8 ± 9.4% and 100.7 ± 1.27% of control respectively, n = 5) (Figure 4C and D).

### The effect of TRPA1 activation on membrane potential

**Figure 5:**
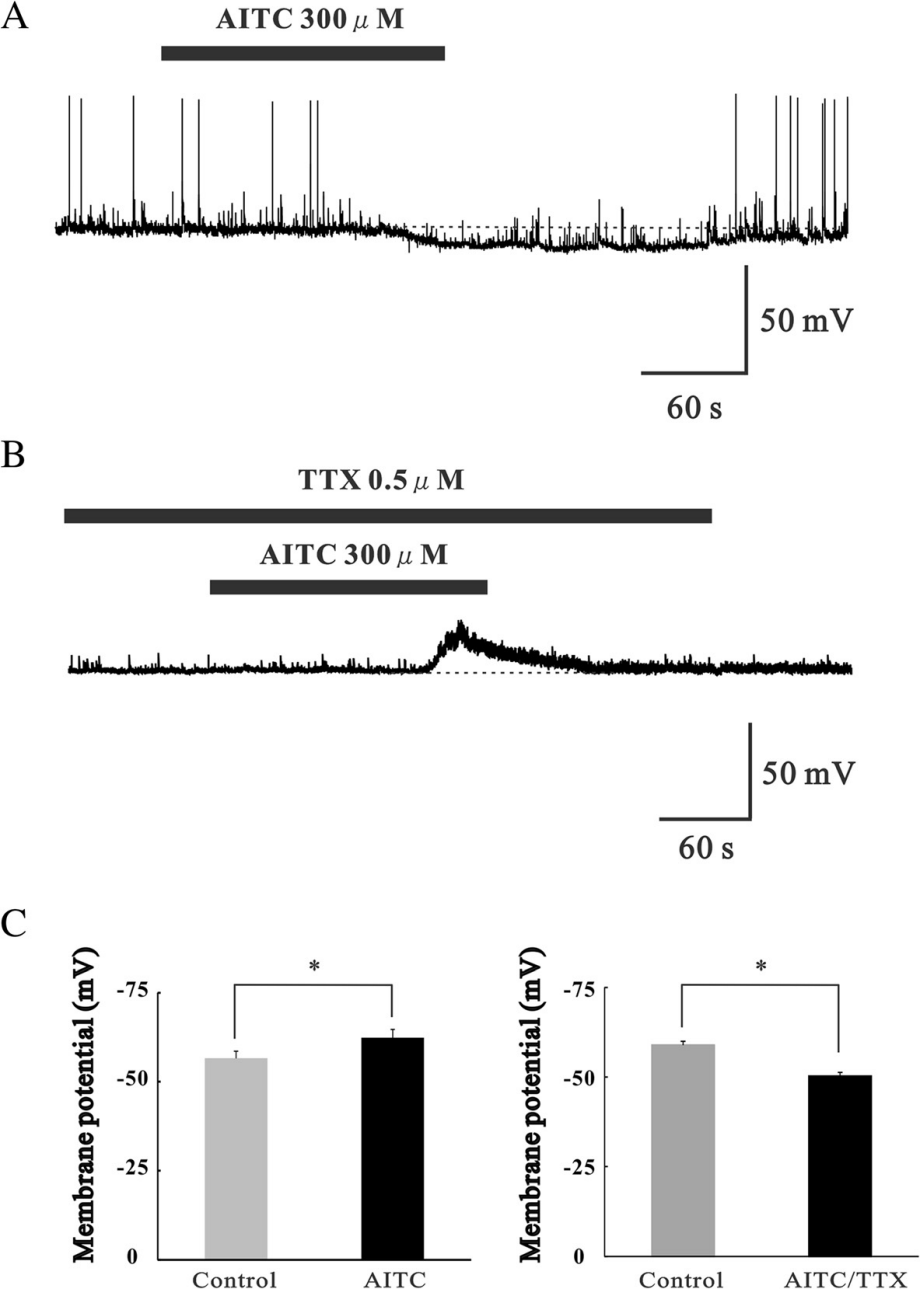
Changes in membrane potential produced by TRPA1 activation in SG neurons. **(A)** A continuous current-clamp recording of the membrane potential showing that AITC (300 μM) evokes hyperpolarization. **(B)** A continuous current-clamp recording of the membrane potential showing that AITC (300 μM) evokes depolarization in the presence of TTX. **(C)** Membrane potential of SG neurons under the action of AITC (n = 5) relative to control (left). Membrane potential of SG neurons under the action of AITC in the presence of TTX (n = 5) relative to control (right). *P < 0.05.

We investigated the effect of TRPA1 activation on membrane potential using *in vivo* patch-clamp methods. The membrane potential was recorded in current-clamp mode with an average recorded from 12 neurons in −59.3 ± 1.4 mV (range: −50.2 to −70.0 mV). Superfusing AITC (300 μM) for 3 min resulted in an increased hyperpolarization in the membrane potential, as shown in Figure 5A. The average change in membrane potential induced by AITC was −5.8 ± 0.7 mV (n = 5; *p* < 0.01) (Figure 5C). While AITC induced depolarization of the membrane potential in the presence of TTX, as shown in Figure 5B, in the presence of TTX, the average change in membrane potential induced by AITC was 6.7 ± 1.3 mV (n = 6; *p* < 0.01) (Figure 5C).

### Effect of AITC on responses to noxious and innocuous stimuli

**Figure 6:**
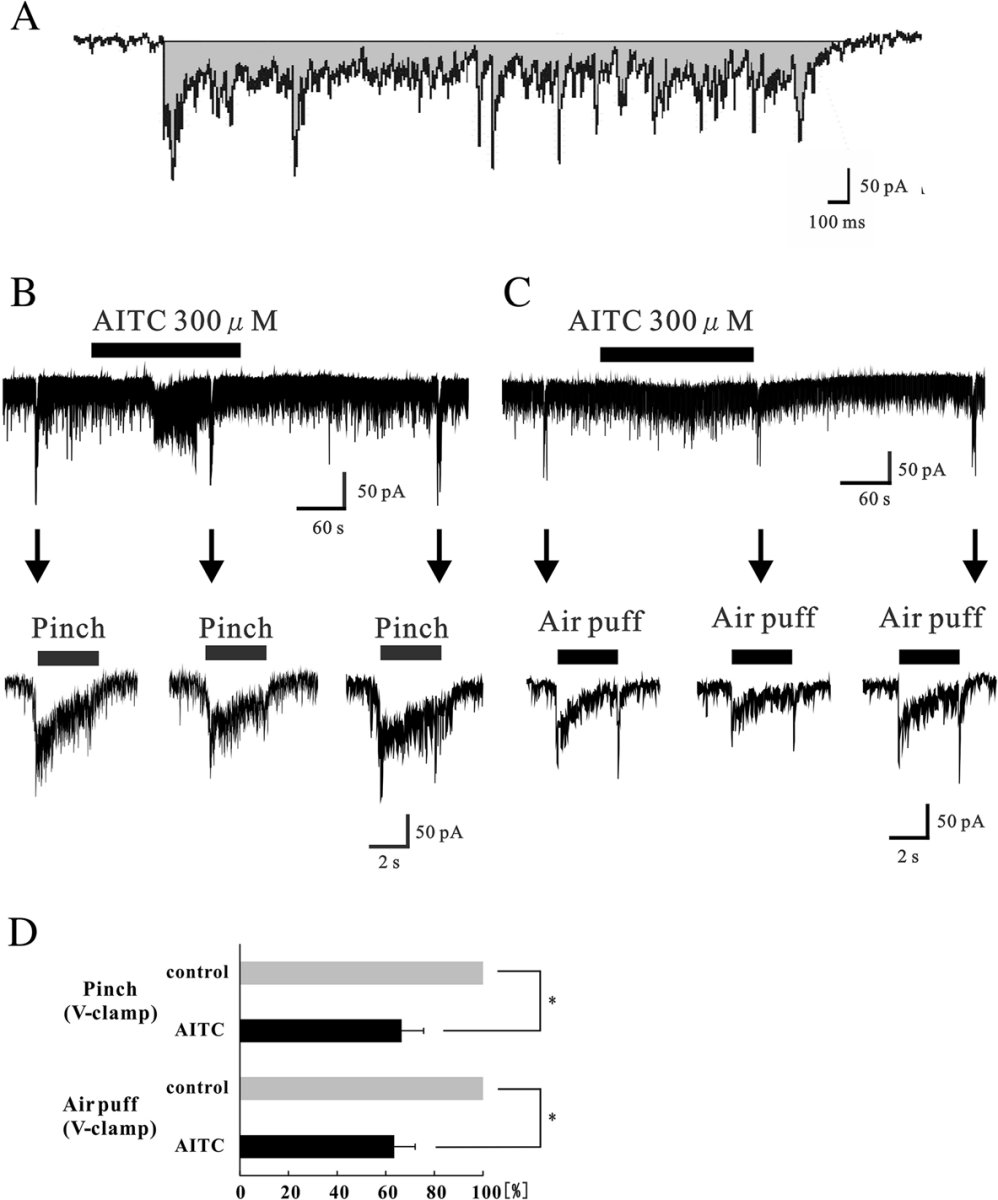
Analysis of the response to noxious and innocuous mechanical stimuli in the presence of AITC in the normal rat. **(A)** Schematic diagram showing the area analyzed. Analysis of the area subtended by the baseline and border of EPSCs was done using software (Clampfit 10, Molecular Devices). **(B)** EPSCs elicited by pinching in voltage-clamp mode (*V*_H_ = −70 mV) in control (left). AITC (300 μM) suppressed repeated EPSCs during pinch (middle), and the barrage of EPSCs induced by pinch appeared again after washout of AITC (right), which showed no desensitization. **(C)** EPSCs elicited by air puff in voltage-clamp mode (*V*_H_ = −70 mV) in control (left). AITC (300 μM) suppressed repeated EPSCs during air puff (middle) and the barrage of EPSCs induced by air puff appeared again after washout of AITC (right) **(D)** Analysis of evoked EPSCs with noxious and innocuous stimuli to the ipsilateral hindlimb. The area significantly decreased during AITC perfusion compared with control in the absence of AITC. Similar results were obtained with pinch (n = 11) and air puff (n = 9) to the skin. *P < 0.05.

SG neurons were examined for their responses to noxious (pinch) and innocuous (air puff) mechanical stimuli. In voltage-clamp mode, pinch or air stimuli applied to the ipsilateral hindlimb elicited a barrage of EPSCs, which disappeared within 1 s following termination of the stimuli. The point on the limb most sensitive to stimulation was different for each cell tested. Stimulating the contralateral hindlimb did not elicit any synaptic responses (data not shown). The peak amplitudes were not determined, because multiple summations resulting from the high-frequency bursting of EPSCs made it difficult to obtain an accurate estimation. As a result, we investigated changes in the area encompassed by the baseline and EPSCs (Figure 6A). These were considered significant when the area increased or decreased by more than 10%. The pinch and air puff stimuli initially produced large and summated EPSCs that were followed by a barrage of EPSCs, and followed by large EPSCs at the end of the stimulus (Figure 6B and C) [24,25].

**Figure 7:**
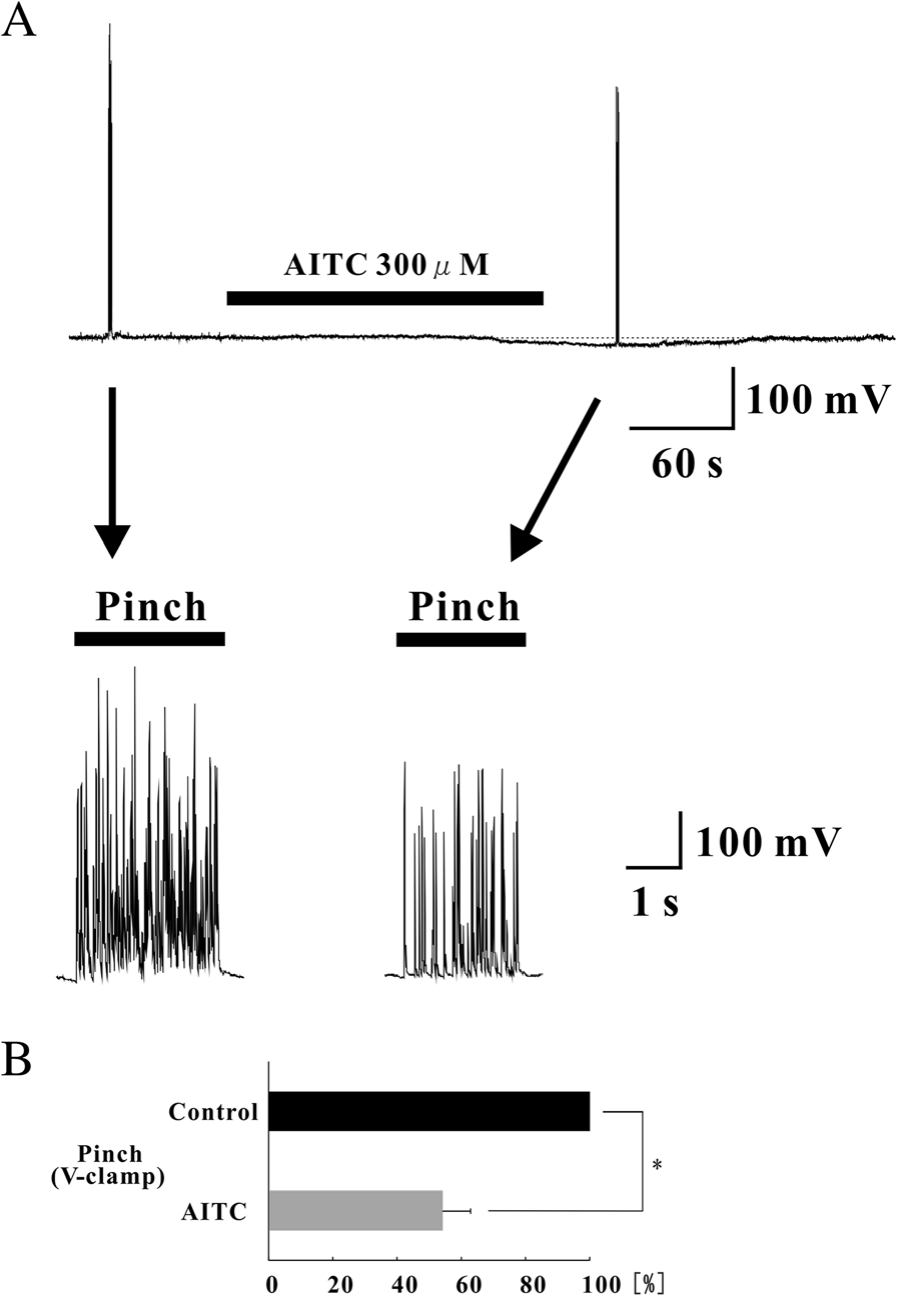
Analysis of the response to pinch stimuli in the presence of AITC under hyperpolarization. **(A)** Continuous chart recording of membrane potential before and during the action of AITC (300 μM; top). Pinch stimuli applied to the ipsilateral hindlimb produced a barrage of EPSPs accompanied by action potentials under a current-clamp condition (bottom left). AITC (300 μM) hyperpolarized the membrane of a SG neuron and inhibited the action potentials (bottom right). **(B)** Analysis of evoked EPSPs and action potentials with noxious stimuli to the ipsilateral hindlimb. The area significantly decreased during AITC perfusion compared with control in the absence of AITC (n = 5). *P < 0.05.

When AITC (300 μM) was applied to the surface of the spinal cord of normal rats, the area of the EPSCs evoked by pinch stimuli significantly decreased in 10 of the 11 neurons tested (66.5 ± 9.1% of control, n = 11; *p* < 0.05) (Figure 6B and D). Similarly, the area of the EPSCs evoked by air puff stimuli also significantly decreased in 6 of the 8 neurons tested (63.5 ± 8.6% of control, n = 8; *p* < 0.05) (Figure 6C and D). The barrage of EPSCs induced by pinch or air puff stimuli appeared again after washout of AITC (Figure 6B and C). We also examined the response to pinch stimuli in current-clamp mode. When AITC (300 μM) was applied to the surface of the spinal cord, the membrane potential hyperpolarized (Figure 7A). In current-clamp mode, the area of the excitatory postsynaptic potentials (EPSPs) and the action potentials evoked by pinch stimuli decreased significantly less than in the absence of AITC (pinch: 54.1 ± 8.7% of control, n = 5; *p* < 0.05) (Figure 7B).

## Discussion

The central terminals derived from primary afferent fibers make their first sensory synapses with spinal dorsal horn neurons. At these synapses, glutamate is used as a fast excitatory neurotransmitter to convey sensory signals from the periphery [26]. The superficial laminae of the spinal dorsal horn, particularly the SG, receive nociceptive information from the viscera, skin and other organs through primary afferent fibers [27]. The pain-sensing channels, including TRP channels, are not only expressed at peripheral nerve endings, but also in central terminals of nociceptive primary afferent fibers innervating SG neurons [28]. TRPA1 is present mainly in small cells in sensory ganglia [13,29,30] and on peptidergic TRPV1-expressing primary sensory neurons [13,31]. It is thought that TRPA1 is upregulated in primary sensory neurons after nerve injury and inflammation [15,32]. In one study, TRPA1 antisense oligodeoxynucleotides decreased the induction of TRPA1 and suppressed inflammation and nerve injury-induced cold hyperalgesia [32]. However, it is unknown how TRPA1 activation of SG neurons influences responses to the noxious stimuli.

In this study, we examined the actions of AITC, an agonist of the TRPA1 channel, on synaptic transmission in SG neurons in the spinal cord using *in vivo* patch-clamp analysis. AITC-induced an increase in EPSC frequency and amplitude that was resistant to TTX. The EPSCs were also suppressed by the addition of CNQX and AP5. This indicates that TRPA1 appears to be localized at presynaptic terminals innervating SG neurons in the spinal cord, and that AITC enhances glutamate release, and the frequency and amplitude of EPSCs in SG neurons, similar to the findings of previous reports [14,22,33]. However, the present study also demonstrated that AITC remarkably increased GABAergic and glycinergic IPSCs in SG neurons. The AITC-induced increase in IPSC frequency and amplitude was abolished in the presence of TTX, as well as with a mixture of CNQX and AP5. Therefore, TRPA1 is likely to be localized not only at presynaptic terminals innervating SG neurons, but also in primary afferent fibers innervating spinal inhibitory interneurons that synapse with SG neurons. These findings are similar to our previous *in vitro* study [14]. However, the TRPV1 agonist capsaicin does not affect inhibitory synaptic transmission in SG neurons [34]. The reason for this is thought to be that the expression of TRPA1 does not appear to fully overlap with that of TRPV1 at the central terminals of primary afferent fibers. Our results could indicate that the activation of TRPA1 has the possibility of antinociceptive actions in the dorsal horn of the spinal cord. A recent study reported that metabolites of acetaminophen activate TRPA1 and have an antinociceptive effect [19]. However, it remains unclear whether the excitatory or inhibitory effect was more robust because TRPA1 activation enhanced both EPSCs and IPSCs in SG neurons. Initially, we analyzed the duration of EPSCs and IPSCs induced by TRPA1 activation to determine this. The duration of enhanced IPSC frequency by TRPA1 activation was significantly longer than that of enhanced EPSC frequency by TRPA1 in SG neurons in this study. Each showed periods of enhanced glutamate release and GABA/glycine release. We interpret the longer duration of the IPSCs to mean that inhibitory interneurons react to TRPA1 fibers activated by AITC more than excitatory neurons. This suggests that the AITC-sensitive pathways may recruit polysynaptic inhibitory inputs onto SG neurons, and that nociceptive transmission may be suppressed by TRPA1 activation on presynaptic terminals of primary afferent fibers in the spinal cord. We also investigated changes in membrane potential, and showed that hyperpolarization occurred in the presence of AITC. However, in presence of TTX, AITC induced depolarization of the membrane potential. These results indicate that the cellular mechanism of AITC-induced hyperpolarization is the summation of enhanced IPSCs, more than EPSCs, by AITC. Taken together, our results suggest that under normal conditions the activation of TRPA1 channels facilitates inhibitory neurotransmission more than excitatory neurotransmission in the dorsal horn, and that the overall effects of TRPA1 activation are antinociceptive.

The activation of TRP channels enhances glutamate release from presynaptic terminals on SG neurons [14,34,35], and TRPA1 and/or TRPV1 activation completely blocks monosynaptic C-fiber-evoked EPSCs *in vitro* [36,37]. Voltage-gated Na^+^ and voltage-gated Ca^2+^ channels are inactivated by the depolarization of C-fiber terminals and axons, and the activation of Ca^2+^-permeable TRP channels causes a robust elevation of Ca^2+^ concentration in C-fiber terminals and enhances EPSCs in SG neurons [38]. These events are thought to block the conduction of action potentials transmitted from the periphery [39]. Similarly, our *in vivo* results suggest that spinal TRPA1 activation decreases the transmission of excitatory input through primary afferent fibers to SG neurons in the spinal cord. It is thought that TRPA1 channels play the role of a low-pass filter and convert excessive high-frequency noxious input through primary afferent fibers into low-frequency activity. In addition, we showed responses to pinch stimuli decrease in the presence of AITC in current-clamp mode. This result indicates that TRPA1 channels play not only the role of a low-pass filter, but also play GABAergic and glycinergic inhibitory roles in the spinal cord, thereby inhibiting excessive pain transmission to the central nervous system.

## Conclusion

In the present study, we demonstrate for the first time that both EPSCs and IPSCs are enhanced by TRPA1 activation in the spinal dorsal horn using *in vivo* patch-clamp methods. We also found that the duration of IPSCs induced by TRPA1 activation are significantly longer than that of EPSCs induced by activation of these channels, and that TRPA1 activation hyperpolarized the membrane potential. Furthermore, we show for the first time that the nociceptive transmission of peripheral tissue stimuli is attenuated by TRPA1 activation under normal conditions. Collectively, our findings suggest that spinal TRPA1 activation may have an antinociceptive effect under normal conditions.

## Methods

All experimental procedures involving the use of animals were approved by the Ethics Committee on Animal Experiments, Wakayama Medical University, and were in accordance with the UK Animals (Scientific Procedures) Act, 1986, and associated guidelines.

### Surgical procedure

The methods used for *in vivo* patch-clamp recordings were similar to those described previously [22-24,39]. Male Sprague–Dawley rats (5 weeks of age, 150–180 g) were anesthetized with urethane (1.2–1.5 g/kg, intraperitoneally). Artificial ventilation of the pneumothorax was not performed as the rats could be maintained in good condition by supplying oxygen through a nose cone [23]. If a withdrawal reflex occurred, a supplemental dose of urethane was given during surgery and the data collection period. A heating pad was placed beneath the rat to maintain body temperature. The lumbar spinal cord was exposed from L3 to L5 following thoraco-lumbar laminectomy from Th12 to L2, and the rat was placed in a stereotaxic apparatus (Model STS-B & SR-5R-HT, Narishige, Tokyo, Japan). Under a binocular microscope with × 8–40 magnification, the dura was cut and removed. The dorsal root that enters the spinal cord above the level of the recording site was gently moved bilaterally using a small glass retractor to expose Lissauer’s tract so that a recording electrode could be advanced into the SG from the surface of the spinal cord. The pia-arachnoid membrane was removed using microforceps to make a window large enough to allow the patch electrode to enter the spinal cord. The surface of the spinal cord was irrigated with 95% O_2_/5% CO_2_-equilibrated Krebs solution (10–15 mL/min; mM: NaCl 117, KCl 3.6, CaCl_2_ 2.5, MgCl_2_ 1.2, NaH_2_PO_4_ 1.2, glucose 11 and NaHCO_3_ 25) through a glass pipette at 36.5 ± 0.5°C. At the end of the experiment, the rats were given an overdose of urethane and then sacrificed by exsanguination.

### Patch-clamp recordings

The patch-electrodes were pulled from thin-walled borosilicate glass capillaries (OD: 1.5 mm) using a P-97 puller (Sutter Instrument, Novato, CA, USA). For whole-cell recordings of EPSCs, electrodes were filled with a patch-pipette solution composed of the following (mM): potassium gluconate 135, KCl 5, CaCl_2_ 0.5, MgCl_2_ 2, EGTA 5, ATP-Mg 5 and HEPES-KOH 5; pH 7.2 (305 mOsm). Recording of IPSCs was performed using an electrode solution composed of the following (mM): Cs_2_SO_4_ 110, tetraethylammonium 5, CaCl_2_ 0.5, MgCl_2_ 2, EGTA 5, ATP-Mg 5 and HEPES-KOH 5; pH 7.2 (305 mOsm). The electrode, with a resistance of 8–12 MΩ, was advanced at an angle of 30–45° into the SG through a window in the pia-arachnoid membrane using a micromanipulator (Model MWS-32S, Narishige). A tight seal (resistance of at least 10 GΩ) was then formed with neurons at a depth of 30–150 μm. Membrane potentials were held at −70 mV in voltage-clamp mode. After forming the seal, the membrane patch was ruptured by a brief period of more negative pressure, thus resulting in a whole cell configuration. Signals were collected using an Axopatch 200B amplifier in conjunction with a Digidata 1440A A/D converter (Molecular Devices, Sunnyvale, CA, USA) and stored on a personal computer using the pCLAMP 10 data acquisition program (Molecular Devices). Recordings were analyzed using Mini Analysis 6.0 software (Synaptosoft, Fort Lee, NJ, USA) and pCLAMP 10. Membrane potential recordings were made in current-clamp mode.

### Stimulation protocols

The methods used for peripheral tissue stimulation were similar to those described previously [24]. Noxious and innocuous mechanical stimuli were applied to the receptive field of the ipsilateral hindlimb using toothed forceps or air puffs (Pressure system IIe, Toohey Company, Fairfield, NJ, USA), respectively. To maintain fixed-strength noxious stimulation, the toothed forceps were clamped during skin pinching. Synaptic responses in SG neurons from animals stimulated with air puffs did not differ between those given a preceding pinch stimulus and those not given a preceding pinch stimulus.

### Drug application

The drugs used in this study were AITC, CNQX, AP5, bicuculline and strychnine. AITC, CNQX, bicuculline and strychnine were first dissolved in dimethyl sulfoxide at 1,000 × the concentration to be used, while AP5 and TTX were dissolved in distilled water at 1,000 × final concentration. All drugs were then diluted to final concentrations in Krebs solution immediately before use and applied by perfusion via a three-way stopcock without any change in perfusion rate or temperature. The time necessary for the solution to flow from the stopcock to the surface of the spinal cord was approximately 40 s.

### Immunohistochemistry

Rats, from which recordings had been made, were perfused through the ascending aorta with saline followed by 4% paraformaldehyde with 1.5% picric acid in 0.16 M phosphate buffered saline (PBS), pH7.2–7.4 (4°C). After fixation, spinal cords were removed and the L4-L5 spinal cord segments were dissected. Spinal cord slices were stained with neurobiotin to label neurons in the SG. Neurobiotin was detected using the avidin-biotin complex (ABC) system (Elite ABC kit; Vector Laboratories, Burlingame, CA, USA). The sections were rinsed in PBS with 0.3% Triton X-100 and 1% normal bovine serum, then incubated with ABC solution diluted with PBS for 2 hours. After three 20-minute rinses in PBS, the sections were reacted with 0.05% diaminobenzidine (DAB) and 0.003% H_2_O_2_ in PBS to visualize injected neurons. After washing, staining was observed by microscopy (Olympus, Tokyo, Japan).

### Statistical analysis

All numerical data were expressed as the mean ± S.E.M. For electrophysiological data, *n* refers to the number of neurons studied. For analysis of the change in frequency and amplitude of postsynaptic currents following the application of AITC, the time course of postsynaptic current frequency before and after AITC application was first constructed with a time bin of 30 s using the Mini Analysis Program 6.0 (Synaptosoft, Decatur, GA). The average response in 30 s of the peak was then used to calculate the percentage change from control. *P* < 0.05, evaluated using Student’s *t*-test or Student’s paired *t*-test, was considered to indicate statistical significance. Similar to previous studies [36,40], cells were deemed to be responsive to the testing compounds when there was a > 20% decrease or increase in the frequency of EPSCs or IPSCs. The Kolmogorov-Smirnov test was used to compare the cumulative distributions of the postsynaptic current parameters in the absence and presence of the test drugs. The membrane potentials were not corrected for the liquid junction potential between the Krebs and patch-pipette solutions.
